# Unilateral total hip replacement patients with symptomatic leg length inequality have abnormal hip biomechanics during walking

**DOI:** 10.1016/j.clinbiomech.2015.02.014

**Published:** 2015-06

**Authors:** Junyan Li, Anthony B. McWilliams, Zhongmin Jin, John Fisher, Martin H. Stone, Anthony C. Redmond, Todd D. Stewart

**Affiliations:** a Department of Design Engineering, School of Science and Technology, Middlesex University, UK; b Leeds Institute for Rheumatic and Musculoskeletal Medicine, School of Medicine, University of Leeds, UK; c NIHR Leeds Biomedical Research Unit, Leeds Teaching Hospitals Trust, Leeds, UK; d Institute of Medical and Biological Engineering, School of Mechanical Engineering, University of Leeds, UK; e School of Mechanical Engineering, Xi'an Jiaotong University, PR China; f Leeds Teaching Hospitals Trust, Chapel Allerton Hospital, Leeds, UK

**Keywords:** Leg length inequality, Hip, Contact force, Arthroplasty, Gait analysis

## Abstract

**Background:**

Symptomatic leg length inequality accounts for 8.7% of total hip replacement related claims made against the UK National Health Service Litigation authority. It has not been established whether symptomatic leg length inequality patients following total hip replacement have abnormal hip kinetics during gait.

**Methods:**

Hip kinetics in 15 unilateral total hip replacement patients with symptomatic leg length inequality during gait was determined through multibody dynamics and compared to 15 native hip healthy controls and 15 ‘successful’ asymptomatic unilateral total hip replacement patients.

**Finding:**

More significant differences from normal were found in symptomatic leg length inequality patients than in asymptomatic total hip replacement patients. The leg length inequality patients had altered functions defined by lower gait velocity, reduced stride length, reduced ground reaction force, decreased hip range of motion, reduced hip moment and less dynamic hip force with a 24% lower heel-strike peak, 66% higher mid-stance trough and 37% lower toe-off peak. Greater asymmetry in hip contact force was also observed in leg length inequality patients.

**Interpretation:**

These gait adaptions may affect the function of the implant and other healthy joints in symptomatic leg length inequality patients. This study provides important information for the musculoskeletal function and rehabilitation of symptomatic leg length inequality patients.

## Highlights

• Gait of symptomatic leg length inequality patients after total hip replacement
• Symptomatic patients' gait more abnormal than asymptomatic patients
• Gait adaptions may affect function of implant and other healthy joints of patients

## 1 Introduction

Leg length inequality (LLI) following total hip replacement (THR) was rarely recognised when the technique was popularised in the 1960s (Charnley, 1979). LLI has come to prominence over the last 20 years due to increasing patient expectations, larger numbers of THRs being performed and the use of THR in a younger and more demanding patient population (Clark et al., 2006; Ellams et al., 2010; McWilliams et al., 2013). One reason for postoperative LLI is malposition of the stem or acetabular components. For example, a stem that is not fully seated may result in lengthening. The incidence of leg length inequality is difficult to ascertain but it has been suggested that some lengthening occurs in as many as 30% of patients following THR (Edeen et al., 1995; Wylde et al., 2009). Due to the multi-factorial nature of complications after total hip replacement and the fact that morbidity is not universal even in the presence of significant LLI, the true incidence of symptomatic LLI remains unclear however (Hofmann and Skrzynski, 2000; Plaass et al., 2011). Although there remains some controversy regarding its clinical significance (White and Dougall, 2002), symptomatic LLI accounts for 8.7% of THR-related claims made against the UK National Health Service Litigation authority (McWilliams et al., 2013). Complaints relating to postoperative LLI are reportedly the most common cause of orthopaedic litigation in the USA (Hofmann and Skrzynski, 2000).

Postoperative symptomatic LLI may lead to abnormal posture, lower back pain and osteoarthritis in the opposite hip and knee and rarer complications such as nerve palsy (Abraham and Dimon, 1992; Clark et al., 2006; Cummings et al., 1993; Giles and Taylor, 1981; Golightly et al., 2009, 2010; McGregor and Hukins, 2009). A shoe raise with physiotherapy is usually adopted as an initial treatment option, although the outcome is not always satisfactory and revision surgery can be necessary if the patient remains symptomatic. Minimising leg length following total hip replacement requires assessment and restoration of the centre of femoral rotation, the femoral offset, and positioning of the acetabular and femoral components (Charles et al., 2005; Clark et al., 2006; Lakshmanan et al., 2008).

As walking is the most common activity of daily living, gait analysis is of value in the evaluation of the functional performance and postoperative rehabilitation in artificial/natural joints, and has been widely used in patients following orthopaedic surgery for research purposes. It has been shown that the walking kinematics and kinetics of THR patients do not return to completely normal values. There is evidence for reduced overall mobility, slower walking speed, altered muscle activity and a less dynamic pattern of kinetics demonstrated in THR patients (Foucher et al., 2007; Li et al., 2014b; Long et al., 1993; Madsen et al., 2004; McCrory et al., 2001; Perron et al., 2000), even in patients with good clinical outcomes. The additional effect of postoperative LLI in THR patients remains less clear. Many kinematic studies of people with LLI have focused on the effects of shoe lifts (Brand and Yack, 1996; Gurney et al., 2001; Walsh et al., 2000) and have concentrated on congenital LLI (Mattei et al., 2013; Perttunen et al., 2004). Having grown up accommodating a lifelong LLI, it cannot be assumed that congenital LLI cases will have the same motion patterns and joint loading as those with a late-onset joint disease who have acquired more recent postoperative LLI following total hip replacement surgery. One previous study of patients with small magnitudes of postoperative LLI (< 10 mm) found little effect on gait kinematics (Rosler and Perka, 2000), although this does not reflect the population of patients with symptomatic LLI who may be considered for revision.

Contact forces can be affected by gait patterns and altered joint contact forces have been linked with potential wear, damage and loosening of implants (Barbour et al., 1995; Cheal et al., 1992; Foucher et al., 2009; Lenaerts et al., 2009; Liu et al., 2008; Phillips et al., 1991; Weber et al., 2012; Williams et al., 2006). It is plausible that patients with potentially pathological magnitudes of postoperative LLI may demonstrate altered gait patterns, and resulting altered hip contact force, relative to healthy controls or indeed relative to asymptomatic THR patients without postoperative LLI.

The aims of the current study were to investigate any alterations in hip kinetics, particularly in hip contact forces, during gait in unilateral THR patients with symptomatic LLI and to compare these to both normal healthy controls (native hips) and to a control group of ‘successful’ unilateral THR patients who had no symptoms. The parameters measured included the ranges of motion and ground reaction force. Moments and joint contact force were then modelled using a multibody dynamics approach and the symmetry of these parameters compared between the operated and non-operated sides, as well as between groups.

## 2 Methods

### 2.1 Subjects

Three cohorts comparable in age and body mass index (BMI) were recruited. All subjects gave written informed consent and the study received local ethical committee approval. A group of 15 patients were recruited who had persisting symptomatic LLI of more than 12 months duration following THR surgery that was severe enough for them to be considered for revision surgery (LLI cohort). Additionally, 15 asymptomatic THR patients of more than 12 months post-THR duration were recruited (THR cohort). All the THR surgeries were performed by one surgeon. As a healthy control group with native hips, 15 healthy normal people (normal cohort) were also recruited.

The case ascertainment for the LLI patient cohort was based initially on the presenting complaint at a regional referral centre, whereby LLI patients complained of having symptoms or functional impairment secondary to a perceived leg length inequality. The presence of LLI in these patients was subsequently confirmed by plain film radiography. The THR cohorts were asymptomatic cases from outpatient department review clinics. Clinical measurements were conducted in all the three cohorts to determine the distance between the anterior superior iliac spine and the medial malleolus, as well as the distance between the greater trochanter and the floor. Subsequent radiographic measurements were conducted for the LLI patients using a previously validated method whereby the inequalities of stem, cup and overall position were measured separately (McWilliams et al., 2011).

### 2.2 Gait analysis

Hip kinematics was acquired for all cases using an eight camera, passive marker system (Vicon MX with T40 cameras, Oxford Metrics, UK) capturing at 150 Hz and 2 megapixel resolution. A 14 marker plug in gait model was used employing 9 mm markers attached to the pelvis, thigh, shank and foot as well described previously (Walsh et al., 2000), and the technical error for this setup within a working volume of 10 x 11 x 2.5 m was calculated as less than 0.2 mm. Kinematic data were integrated with force plate data from two Bertec force plates (Bertec Corp, Worthington, OH), capturing at 1000 Hz in a 5 m walkway. All subjects walked at a self-determined normal speed. Following an acclimatisation period, gait data were acquired from three passes along an 8 m walkway with clean strikes on the force plates observed. Gait kinematic data were tracked and pre-processed using Vicon Nexus version 1.7 and ground reaction force data were integrated and exported in C3D format.

### 2.3 Biomechanical analysis

Muscle forces and the resultant hip contact force were determined using commercial multibody dynamics software (AnyBody, version 5.0, AnyBody Technology, Aalborg, Denmark). The calculations were based on inverse dynamics in which the muscle and joint forces are calculated from the external moments that can be directly derived from the measured kinematics and ground reaction force. The musculoskeletal model in the AnyBody Repository is based on an anthropometric dataset provided by the University of Twente (Horsman and Dirk, 2007) and has been validated previously for predicting hip contact forces. (Forster, 2004; Manders et al., 2008). The model comprises of a whole human lower extremity including 340 muscles and 11 rigid bodies representing talus, foot, shank, patella and thigh for both legs and the pelvis. The model was scaled to reflect joint positions and body sizes for each participant. The model was asymmetrical so that the LLI of the patients can be reflected in the scaled model. Since the musculoskeletal model is a redundant system in which there are more muscles forces than necessary to balance the external moments/forces, unique solutions cannot be achieved. To solve this problem, a physiologically feasible optimization approach was performed through minimising the sum of muscle stresses squared (Glitsch and Baumann, 1997; Heintz and Gutierrez-Farewik, 2007).

Hip contact forces were calculated, along with external moments and rotational angles of the hip and ground reaction forces. The forces and moments were presented as the total magnitude to facilitate the comparison to previous literature due to the potential differences in coordinate systems used by different studies. Forces were normalised to body weight (BW) and moments were normalised to BW and height (Ht) to offset the variations in BW and Ht among subjects. In addition, gait symmetry in all the recorded parameters was determined through calculation of the absolute value of the difference between the two limbs of each participant for the three cohorts, in order to explore the functional consequences of limping in the cohorts.

### 2.4 Statistical analysis

Mean values, along with the associated 95% confidence intervals (CI) were calculated to quantify the variation within each group. Generally, the hip contact force during gait can be characterised by a 1st peak (~ 15% gait cycle associated with heel-strike/weight acceptance) to a trough (~ 32% gait cycle near mid-stance) and then to a 2nd peak (~ 50% gait cycle prior to toe-off). These three events are often referred to as F_1_, F_2_ and F_3_. For this reason, inferential statistical analyses as described below were performed to explore systematic differences at each of the time points F_1–3_. Since some of the gait data were not normally distributed, non-parametric statistical tests were used. The presence of any systematic differences between cohorts was determined through Mann–Whitney tests. Spearman correlations were performed to explore the relationship between the hip contact forces and the magnitude of LLI (stem position, cup position and overall position) for the LLI cohort. Additionally, joint forces on the operated limb and non-operated limb were compared using a Wilcoxon test to investigate any systematic differences in hip contact force between the operated and non-operated limbs. A significance level of *P* < 0.05 was regarded as significant throughout. All statistical analyses were performed using SPSS v 18.0 (SPSS Inc., Chicago, IL).

## 3 Results

The symptomatic LLI patients had comparable age and BMI to the asymptomatic THR patients and normal healthy individuals (Table 1). As might be expected, the clinical measurements indicated substantially greater magnitudes of LLI in the symptomatic LLI patients compared to the asymptomatic THR patients and normal healthy individuals.

**Fig. 1:**
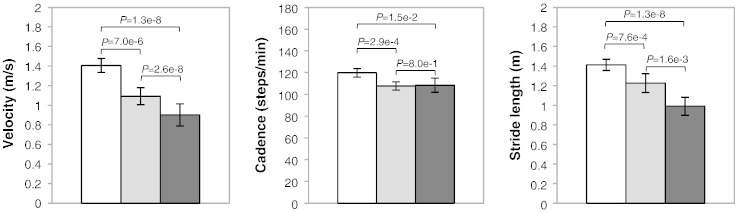
Mean (95% CI) for gait velocity, cadence and stride length in symptomatic LLI patients (black), compared to normal healthy individuals (white) and to asymptomatic THR patients (grey).

The gait velocity, cadence and stride length for the three cohorts are shown in Fig. 1. Velocity, cadence and stride length were significantly reduced in the LLI and THR cohorts, compared with the normal controls. The LLI cohort had significantly decreased velocity and stride length, but comparable cadence in comparison to the THR patients.

**Fig. 2:**
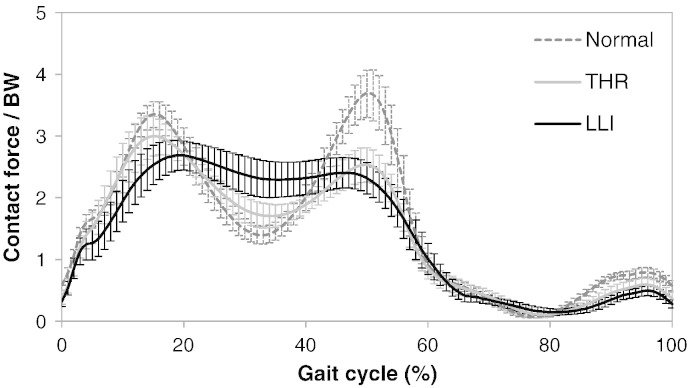
Hip contact force (mean and 95% CI) during a gait cycle for the symptomatic LLI patients compared to the normal individuals and the asymptomatic THR patients. Both post-operative groups differed from the controls, with a systematic trend towards greater alteration in hip contact forces in the LLI group compared to the asymptomatic THR patients.

The mean hip contact force for the three cohorts is shown in Fig. 2. Different loading patterns were found in the three cohorts. The characteristic peak–trough–peak pattern in hip contact force, as observed in the normal healthy controls and to a lesser extent in the asymptomatic THR patients, was reduced further in the LLI group. Compared with the normal individuals, the LLI patients exhibited a 24% lower F_1_ (*P* = 0.00058), 66% higher F_2_ (*P* < 0.0001) and 37% lower F_3_ (*P* < 0.0001) of hip contact force. In comparison to the THR patients, the LLI cohort had comparable heel-strike (*P* = 0.11) and toe-off (*P* = 0.33) peaks but a 34% higher mid-stance trough (*P* = 0.005).

**Fig. 3:**
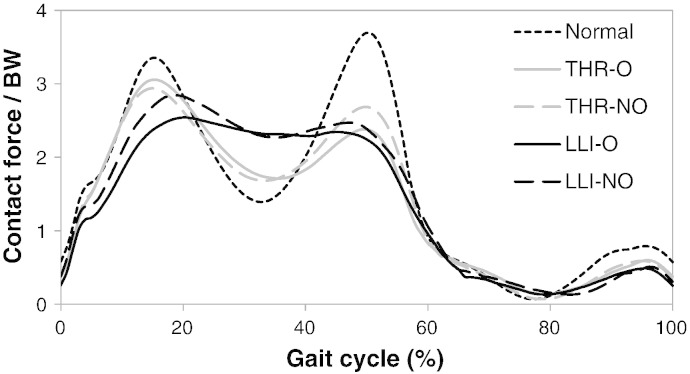
Mean hip contact forces for the operated (O) and non-operated (NO) limbs of the LLI and THR cohorts in comparison to the normal individuals. Alteration of hip contact force was seen in both the operated and non-operated limbs in the THR and LLI patients.

The mean hip contact force for the operated and non-operated limbs in the LLI and THR patients, along with that of the normal individuals, is shown in Fig. 3. Compared with the normal cohort, alteration in hip loads was found for both the operated and non-operated limbs of the LLI and THR patients. Differences between operated and non-operated limbs were not significantly different however, in either the LLI or THR patients (*P* > 0.05).

**Fig. 4:**
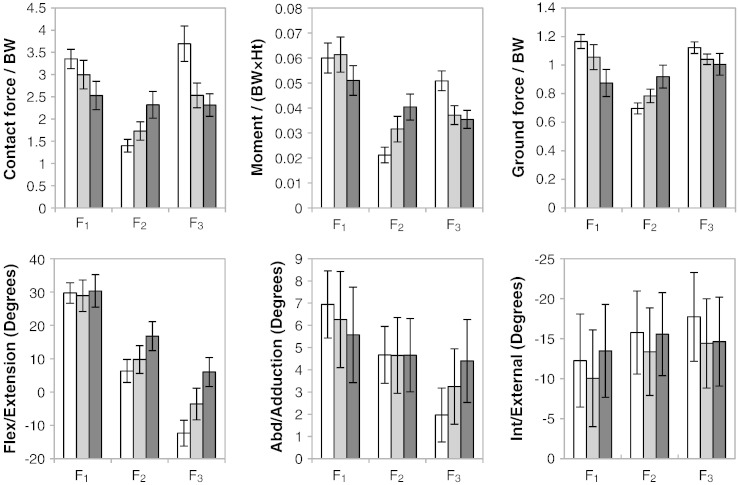
Summary (mean and 95% CI) of hip contact force, moment, ground reaction force and range of motion at F_1–3_ for normal healthy individuals (white), asymptomatic THR patients (grey) and symptomatic LLI patients (black). Compared with THR patients, the LLI patients had greater abnormalities in hip force, moment, range of motion and ground reaction force.

The hip contact force, moment, ground reaction force and range of motion at each of the points F_1–3_ are summarised in Fig. 4 for the three cohorts. For all of these parameters, the THR patients exhibited different F_1–3_ patterns compared with the normal cohort, and a greater magnitude of variation from the normal group was found in the LLI patients compared to the THR group. Besides the joint force alterations mentioned previously, significant abnormalities within the LLI cohort included: lower flexion moment (*P* = 0.05) and ground reaction force (*P* < 0.0001) at heel-strike; higher flexion moment (*P* < 0.0001), ground reaction force (*P* < 0.0001) and flexion/extension angle (*P* = 0.002) at mid-stance; lower flexion moment (*P* < 0.0001), ground reaction force (*P* = 0.001) and higher flexion/extension (*P* < 0.0001) at toe-off.

**Fig. 5:**
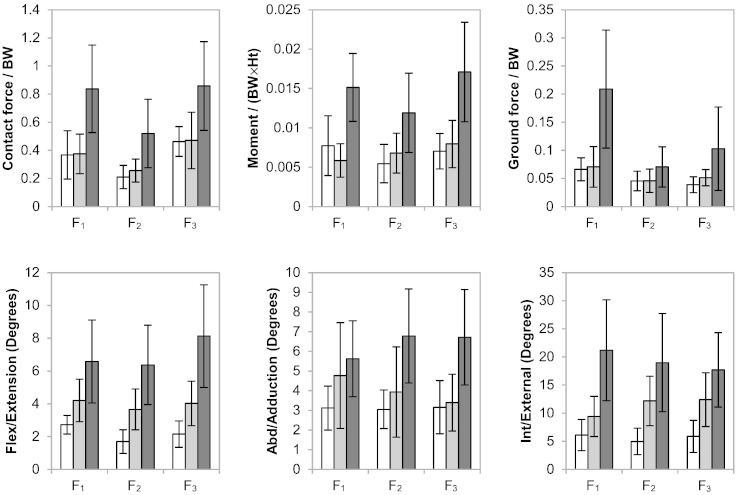
Summary (mean and 95% CI) of symmetry in hip contact force, moment, ground reaction force and range of motion (magnitude of between-side differences) at F_1–3_ for the normal healthy individuals (white), asymptomatic THR patients (grey) and symptomatic LLI patients (black). Compared with THR patients, the LLI patients had greater asymmetries in hip force, moment, range of motion and ground reaction force.

As shown in Fig. 5, Magnitudes of hip contact force, moment and ground reaction force were relatively symmetrical between operated and non-operated sides in the normal and THR cohorts and between-side differences did not approach statistical significance, whilst the LLI patients exhibited markedly greater asymmetry. Significant greater asymmetry within the LLI cohort as compared to the normal cohort included: hip contact force (*P* = 0.029), moment (*P* = 0.013), flexion/extension angle (*P* = 0.012) and internal/external rotation angle (*P* = 0.0011) at heel-strike; moment (*P* = 0.023), flexion/extension angle (*P* = 0.0059), abduction/adduction angle (*P* = 0.026) and internal/external rotation angle (*P* = 0.0001) at mid-stance; moment (*P* = 0.011), flexion/extension angle (*P* = 0.0001), abduction/adduction angle (*P* = 0.029) and internal/external rotation angle (*P* = 0.0019) at toe-off.

The plain film radiographic findings confirmed the clinical measures in the LLI group with each of the patients having at least one of the three LLI parameters greater than 10 mm, and therefore demonstrating a structural LLI (Table 2). No systematic relationships were identified between the magnitude of LLI and absolute hip contact forces, or between magnitude of LLI and symmetry of hip contact force.

## 4 Discussion

Functional impairment can persist postoperatively, particularly when the outcome of the surgical procedure is technically sub-optimal even when due to no fault of the surgeon. Stratified investigations, aimed at better understanding sub-groups of patients, are necessary to enhance our understanding of the postoperative outcomes of patients and the implications for implant longevity.

In the current study, the mean peak hip contact force for our normal individuals was 3.97 BW which agrees well with previous reports for normal cohorts such as 3.89 BW by Paul (1967). In addition, the average peak hip force for the THR and LLI patients were 3.16 BW and 3.25 BW respectively, which are within the range published in the literature for THR patients (2.4 BW to 4.1 BW) (Bergmann et al., 1993, 2001; Brand et al., 1994; Davy et al., 1988; Kotzar et al., 1991).

The relatively small between-subject variability within each group suggests that factors associated with simply having undergone THR and also factors arising as a consequence of having a persisting LLI resulted in systematic changes to the gait pattern. One of the more significant findings is that the LLI patients had a greater difference from the control group in hip biomechanics than the asymptomatic THR patients. Although any reduction in hip force could be attributed to slower walking speeds, the varying hip contact forces between the asymptomatic THR patients and LLI patients are unlikely to be simply the result of their different walking speeds. These two cohorts had marked different hip contact forces during stance phase, although their hip forces during heel-strike and toe-off were of similar magnitude. The diminished peak–trough–peak dynamic pattern and the significantly higher magnitude of joint contact force around mid-stance, along with the altered walking speed, may pose a specific concern for the LLI patients. The dynamic nature of natural joint motion with a lower swing phase load and a faster joint motion, duplicated by a well-functioning THR, may encourage improved lubrication mechanisms and a reduced contact area, whereas a relatively flat curve may suppress squeeze-film action, migrating contact area effect and lubricant entrainment, potentially accompanied by larger contact areas resulted from the increased swing phase loads (Jin, 2006). Whilst the precise consequences will require further elucidatory work, the immediate implication is that joint contact forces appear to vary in subgroups of THR patients and that these variations are not currently incorporated into design or testing of implants. The results thus stress the importance of evaluating bearings in vitro or in silico under the broader range of tribological conditions that may be expected in vivo.

Gait alterations were also found for the non-operated limbs of the LLI patients, with reduced peak loads and increased mid-stance joint force. As the properties and strength of the joint cartilage tend to adapt to regular level of stress (Arokoski et al., 1999), the less dynamic joint load with reduced magnitude may, in the long term, alter the ultrastructure of the cartilage on the non-operated healthy hips for the LLI patients, potentially leaving healthy hips more vulnerable to insults involving sudden high loads (Li et al., 2014a, 2014c; Swann and Seedhom, 1993).

Compared with the asymptomatic THR patients, the poorer functional performance of the LLI patients is also reflected in their greater asymmetry, the result of which is that patients are more likely to notice and complain about LLI. Interestingly, neither the absolute hip contact force nor the between-side symmetry was correlated with the magnitude of LLI however. This suggests that the difference between the THR and LLI patients in hip biomechanics, cannot be simply explained by the amount of LLI, and that there are other more complex interactions involved, such as anatomical variations and availability of compensation mechanisms. Preoperative adaptation has been proposed to be another reason for the alteration in postoperative gait pattern (Foucher et al., 2007). Further work is required to establish whether the reduced contact forces at toe-off observed in the THR and LLI groups are a consequence of the joint replacement surgery or a trait acquired pre-operatively from habituation to an antalgic gait.

Regarding the potential hazards to the implant and other healthy joints, it is clear that attempts should be made both to minimise structural LLI intra-operatively and to optimise postoperative rehabilitation in order to minimise the risk of persisting abnormalities in gait biomechanics as noted in both groups of patients following THR.

In this study, computational musculoskeletal models were constructed to calculate joint forces, because direct experimental measurements are too invasive for use with either healthy joints or patient participants. There are some limitations associated with computational modelling however. In this study, which included relatively large numbers of patients, we did not attempt to scale bony anatomy individually nor to individualise muscle architecture and activation patterns, employing instead the scaling of general parameters built in to the AnyBody model. These simplifications have been reported however as having little influence on joint contact forces (Besier et al., 2003; Carbone et al., 2012) and importantly would be expected to apply equally in all three groups thereby limiting the risk of their contributing to spurious findings of systematic differences. Factors such as skin movement artefact are well recognised limitations when modelling is based on optoelectronic motion tracking data, although in the current study BMI was matched in the THR and LLI groups and so the effect would again be seen equally in both groups. Additionally skin movement artefact has less effect on the measurement of flexion which was the most significant parameter in the study, than it does on long axis rotation or abduction/adduction. Although all the subjects were randomly recruited, sex ratio was not comparable between the cohorts, because females might be less likely to tolerate a given magnitude of LLI due to their pelvis geometry and smaller body size as compared to males. It is unclear to what extent sex distribution may affect the results, which will be further evaluated in future studies.

In conclusion, more significant gait abnormalities were found in our cohort of symptomatic postoperative LLI patients than in asymptomatic THR patients. The LLI patients had poorer function defined by lower gait velocity, reduced stride length, reduced ground reaction force, impaired hip range of motion and reduced hip moment. The less dynamic hip force observed in LLI patients was characterised by significantly reduced peak loads, substantially increased mid-stance loads and a greater asymmetry from heel-strike to toe-off. These adaptions may affect the function and hence the longevity of the implant as in combination they may influence lubricant entrainment, which will be further investigated in the future.

## Conflict of interest

None of the authors have any financial or personal relationships with other people or organisationsthat could have inappropriately influenced or biased the work.

## Figures and Tables

**Table 1:** Mean (95% CI) for number of male/female, age, BMI, LLI from anterior superior iliac spine (ASIS) to medial malleolus (MM) and LLI from greater trochanter (GT) to floor in normal healthy individuals, asymptomatic THR patients and symptomatic LLI patients.

	Male/Female	Age [years]	BMI [kg/mm^2^]	LLI (ASIS to MM) [mm]	LLI (GT to floor) [mm]
Normal	8/7	58 (55 to 61)	24.5 (23.0 to 25.9)	5.7 (3.4 to 8.0)	5.0 (2.1 to 7.9)
THR	11/4	64 (59 to 70)	30.7 (27.7 to 33.8)	10.3 (4.7 to 15.9)	5.3 (2.0 to 8.6)
LLI	3/12	61 (56 to 65)	27.3 (25.7 to 28.9)	20.0 (11.9 to 28.1)	23.0 (15.6 to 30.4)

**Table 2:** Mean and 95% CI (mm) of LLI, and relationships between magnitude and symmetry (magnitude of between-side difference) of hip contact forces and magnitude of LLI for the LLI patients. ρ — Correlation coefficient (1 and − 1 values mean monotonous increase and decrease respectively).

	Cup position	Stem position	Overall
Between-side difference in LLI (95% CI)	13.3 (9.1 to 17.6)	1.1 (− 1.3 to 3.4)	14.4 (8.7 to 20.1)
F_1_	ρ = − 0.08 *P* = 0.78	ρ = − 0.03 *P* = 0.91	ρ = − 0.12 *P* = 0.68
F_2_	ρ = 0.43 *P* = 0.11	ρ = − 0.08 *P* = 0.79	ρ = 0.16 *P* = 0.56
F_3_	ρ = − 0.05 *P* = 0.87	ρ = − 0.32 *P* = 0.24	ρ = − 0.19 *P* = 0.51
F_1_ symmetry	ρ = 0.09 *P* = 0.76	ρ = − 0.01 *P* = 0.98	ρ = − 0.01 *P* = 0.97
F_2_ symmetry	ρ = 0.45 *P* = 0.10	ρ = 0.30 *P* = 0.28	ρ = 0.42 *P* = 0.12
F_3_ symmetry	ρ = 0.27 *P* = 0.33	ρ = − 0.11 *P* = 0.69	ρ = 0.14 *P* = 0.62

## References

[bb0005] Abraham W., Dimon J. (1992). Leg length discrepancy in total hip arthroplasty. Orthop. Clin. N. Am..

[bb0010] Arokoski J.P.A., Hyttinen M.M., Helminen H.J., Jurvelin J.S. (1999). Biomechanical and structural characteristics of canine femoral and tibial cartilage. J. Biomed. Mater. Res..

[bb0015] Barbour P.S.M., Barton D.C., Fisher J. (1995). The influence of contact stress on the wear of UHMWPE for total replacement hip prostheses. Wear.

[bb0025] Bergmann G., Graichen F., Rohlmann A. (1993). Hip joint loading during walking and running, measured in two patients. J. Biomech..

[bb0020] Bergmann G., Deuretzbacher G., Heller M., Graichen F., Rohlmann A., Strauss J., Duda G.N. (2001). Hip contact forces and gait patterns from routine activities. J. Biomech..

[bb0030] Besier T.F., Sturnieks D.L., Alderson J.A., Lloyd D.G. (2003). Repeatability of gait data using a functional hip joint centre and a mean helical knee axis. J. Biomech..

[bb0040] Brand R.A., Yack H.J. (1996). Effects of leg length discrepancies on the forces at the hip joint. Clin. Orthop. Relat. Res..

[bb0035] Brand R.A., Pedersen D.R., Davy D.T., Kotzar G.M., Heiple K.G., Goldberg V.M. (1994). Comparison of hip force calculations and measurements in the same patient. J. Arthroplast..

[bb0045] Carbone V., Van Der Krogt M.M., Koopman H.F.J.M., Verdonschot N. (2012). Sensitivity of subject-specific models to errors in musculo-skeletal geometry. J. Biomech..

[bb0050] Charles M.N., Bourne R.B., Davey J.R., Greenwald A.S., Morrey B.F., Rorabeck C.H. (2005). Soft-tissue balancing of the hip: the role of femoral offset restoration. Instr. Course Lect..

[bb0055] Charnley J. (1979). Low friction arthroplasty of the hip: theory and practice.

[bb0060] Cheal E.J., Spector M., Hayes W.C. (1992). Role of loads and prosthesis material properties on the mechanics of the proximal femur after total hip arthroplasty. J. Orthop. Res..

[bb0065] Clark C.R., Huddleston H.D., Schoch E.P., Thomas B.J. (2006). Leg-length discrepancy after total hip arthroplasty. J. Am. Acad. Orthop. Surg..

[bb0070] Cummings G., Scholz J., Barnes K. (1993). The effect of imposed leg length difference on pelvic bone symmetry. Spine.

[bb0075] Davy D.T., Kotzar G.M., Brown R.H., Heiple K.G., Goldberg V.M., Heiple K.G., Berilla J., Burstein A.H. (1988). Telemetric force measurements across the hip after total arthroplasty. J. Bone Joint Surg. Am..

[bb0080] Edeen J., Sharkey P., Alexander A. (1995). Clinical significance of leg-length inequality after total hip arthroplasty. Am. J. Orthop. (Belle Mead N.J.).

[bb0085] Ellams D., Forsyth O., Mistry A. (2010). National Joint Registry for England and Wales. 7th Annual Report.

[bb0090] Forster E. (2004). Predicting Muscle Forces in the Human Lower Limb During Locomotion.

[bb0095] Foucher K.C., Hurwitz D.E., Wimmer M.A. (2007). Preoperative gait adaptations persist one year after surgery in clinically well-functioning total hip replacement patients. J. Biomech..

[bb0100] Foucher K.C., Hurwitz D.E., Wimmer M.A. (2009). Relative importance of gait vs. joint positioning on hip contact forces after total hip replacement. J. Orthop. Res..

[bb0105] Giles L., Taylor J. (1981). Low-back pain associated with leg length inequality. Spine.

[bb0110] Glitsch U., Baumann W. (1997). The three-dimensional determination of internal loads in the lower extremity. J. Biomech..

[bb0115] Golightly Y., Allen K., Helmick C., Renner J., Jordan J. (2009). Symptoms of the knee and hip in individuals with and without limb length inequality. Osteoarthr. Cartil..

[bb0120] Golightly Y.M., Allen K.D., Helmick C.G., Schwartz T.A., Renner J.B., Jordan J.M. (2010). Hazard of incident and progressive knee and hip radiographic osteoarthritis and chronic joint symptoms in individuals with and without limb length inequality. J. Rheumatol..

[bb0125] Gurney B., Mermier C., Robergs R., Gibson A., Rivero D. (2001). Effects of limb-length discrepancy on gait economy and lower-extremity muscle activity in older adults. J. Bone Joint Surg..

[bb0130] Heintz S., Gutierrez-Farewik E.M. (2007). Static optimization of muscle forces during gait in comparison to EMG-to-force processing approach. Gait Posture.

[bb0135] Hofmann A.A., Skrzynski M.C. (2000). Leg-length inequality and nerve palsy in total hip arthroplasty: a lawyer awaits!. Orthopedics.

[bb0140] Horsman K., Dirk M. (2007). The Twente Lower Extremity Model: Consistent Dynamic Simulation of the Human Locomotor Apparatus.

[bb0145] Jin Z. (2006). Theoretical studies of elastohydrodynamic lubrication of artificial hip joints. Proc. Inst. Mech. Eng. Part J J. Eng. Tribol..

[bb0150] Kotzar G.M., Davy D.T., Goldberg V.M., Heiple K.G., Berilla J., Brown R.H., Burstein A.H. (1991). Telemeterized in vivo hip joint force data: a report on two patients after total hip surgery. J. Orthop. Res..

[bb0155] Lakshmanan P., Ahmed S.M., Hansford R.G., Woodnutt D.J. (2008). Achieving the required medial offset and limb length in total hip arthroplasty. Acta Orthop. Belg..

[bb0160] Lenaerts G., Mulier M., Spaepen A., Van Der Perre G., Jonkers I. (2009). Aberrant pelvis and hip kinematics impair hip loading before and after total hip replacement. Gait Posture.

[bb0165] Li J., Hua X., Jin Z., Fisher J., Wilcox R.K. (2014). Biphasic investigation of contact mechanics in natural human hips during activities. Proc. Inst. Mech. Eng. H J. Eng. Med..

[bb0170] Li J., Redmond A.C., Jin Z., Fisher J., Stone M.H., Stewart T.D. (2014). Hip contact forces in asymptomatic total hip replacement patients differ from normal healthy individuals: implications for preclinical testing. Clin. Biomech..

[bb0175] Li J., Wang Q., Jin Z., Williams S., Fisher J., Wilcox R.K. (2014). Experimental validation of a new biphasic model of the contact mechanics of the porcine hip. Proc. Inst. Mech. Eng. H J. Eng. Med..

[bb0180] Liu F., Leslie I., Williams S., Fisher J., Jin Z. (2008). Development of computational wear simulation of metal-on-metal hip resurfacing replacements. J. Biomech..

[bb0185] Long W.T., Dorr L.D., Healy B., Perry J. (1993). Functional recovery of noncemented total hip arthroplasty. Clin. Orthop. Relat. Res..

[bb0190] Madsen M.S., Ritter M.A., Morris H.H., Meding J.B., Berend M.E., Faris P.M., Vardaxis V.G. (2004). The effect of total hip arthroplasty surgical approach on gait. J. Orthop. Res..

[bb0195] Manders C., New A., Rasmussen J. (2008). Validation of musculoskeletal gait simulation for use in investigation of total hip replacement. 16th Congress of the European Society of Biomechanics. J. Biomech..

[bb0200] Mattei L., Campioni E., Accardi M.A., Dini D. (2013). Finite element analysis of the meniscectomised tibio-femoral joint: implementation of advanced articular cartilage models. Comput. Meth. Biomech. Biomed. Eng..

[bb0205] McCrory J.L., White S.C., Lifeso R.M. (2001). Vertical ground reaction forces: objective measures of gait following hip arthroplasty. Gait Posture.

[bb0210] Mcgregor A.H., Hukins D.W.L. (2009). Lower limb involvement in spinal function and low back pain. J. Back Musculoskelet. Rehabil..

[bb0220] Mcwilliams A., Stewart T.D., Grainger A.J., O'Connor P.J., White D., Redmond A., Stone M.H. (2011). Leg length inequality following total hip replacement. J. Orthop. Trauma.

[bb0215] Mcwilliams A., Douglas S., Redmond A., Grainger A., O'Connor P., Stewart T., Stone M. (2013). Litigation after hip and knee replacement in the National Health Service. Bone Joint J..

[bb0225] Paul J.P. (1967). Forces transmitted by joints in the human body.

[bb0230] Perron M., Malouin F., Moffet H., Mcfadyen B.J. (2000). Three-dimensional gait analysis in women with a total hip arthroplasty. Clin. Biomech..

[bb0235] Perttunen J.R., Anttila E., Södergård J., Merikanto J., Komi P.V. (2004). Gait asymmetry in patients with limb length discrepancy. Scand. J. Med. Sci. Sports.

[bb0240] Phillips T.W., Nguyen L.T., Munro S.D. (1991). Loosening of cementless femoral stems: a biomechanical analysis of immediate fixation with loading vertical, femur horizontal. J. Biomech..

[bb0245] Plaass C., Clauss M., Ochsner P.E., Ilchmann T. (2011). Influence of leg length discrepancy on clinical results after total hip arthroplasty—a prospective clinical trial. Hip Int..

[bb0250] Rosler J., Perka C. (2000). The effect of anatomical positional relationships on kinetic parameters after total hip replacement. Int. Orthop..

[bb0255] Swann A.C., Seedhom B.B. (1993). The stiffness of normal articular cartilage and the predominant acting stress levels: implications for the aetiology of osteoarthrosis. Br. J. Rheumatol..

[bb0260] Walsh M., Connolly P., Jenkinson A., O'Brien T. (2000). Leg length discrepancy — an experimental study of compensatory changes in three dimensions using gait analysis. Gait Posture.

[bb0265] Weber T., Dendorfer S., Dullien S., Grifka J., Verkerke G.J., Renkawitz T. (2012). Measuring functional outcome after total hip replacement with subject-specific hip joint loading. Proc. Inst. Mech. Eng. H J. Eng. Med..

[bb0270] White T., Dougall T. (2002). Arthroplasty of the hip leg length is not important. J. Bone Joint Surg. Br. Vol..

[bb0275] Williams S., Jalali-Vahid D., Brockett C., Jin Z., Stone M.H., Ingham E., Fisher J. (2006). Effect of swing phase load on metal-on-metal hip lubrication, friction and wear. J. Biomech..

[bb0280] Wylde V., Whitehouse S.L., Taylor A.H., Pattison G.T., Bannister G.C., Blom A.W. (2009). Prevalence and functional impact of patient-perceived leg length discrepancy after hip replacement. Int. Orthop..

